# Increased Predictive Accuracy of Multi-Environment Genomic Prediction Model for Yield and Related Traits in Spring Wheat (*Triticum aestivum* L.)

**DOI:** 10.3389/fpls.2021.720123

**Published:** 2021-10-08

**Authors:** Vipin Tomar, Daljit Singh, Guriqbal Singh Dhillon, Yong Suk Chung, Jesse Poland, Ravi Prakash Singh, Arun Kumar Joshi, Yogesh Gautam, Budhi Sagar Tiwari, Uttam Kumar

**Affiliations:** ^1^Borlaug Institute for South Asia, Ludhiana, India; ^2^Department of Biological Sciences and Biotechnology, Institute of Advanced Research, Gandhinagar, India; ^3^International Maize and Wheat Improvement Center, New Delhi, India; ^4^Department of Plant Pathology, Kansas State University, Manhattan, KS, United States; ^5^Department of Biotechnology, Thapar Institute of Engineering & Technology, Patiala, India; ^6^Department of Plant Resources and Environment, Jeju National University, Jeju-si, South Korea; ^7^Global Wheat Program, International Maize and Wheat Improvement Center, Texcoco, Mexico

**Keywords:** single-environment, multi-environments, genotyping by sequencing, genomic selection (GS), genomics predictions, best linear unbiased predictions, wheat

## Abstract

Genomic selection (GS) has the potential to improve the selection gain for complex traits in crop breeding programs from resource-poor countries. The GS model performance in multi-environment (ME) trials was assessed for 141 advanced breeding lines under four field environments *via* cross-predictions. We compared prediction accuracy (PA) of two GS models with or without accounting for the environmental variation on four quantitative traits of significant importance, i.e., grain yield (GRYLD), thousand-grain weight, days to heading, and days to maturity, under North and Central Indian conditions. For each trait, we generated PA using the following two different ME cross-validation (CV) schemes representing actual breeding scenarios: (1) predicting untested lines in tested environments through the ME model (ME_CV1) and (2) predicting tested lines in untested environments through the ME model (ME_CV2). The ME predictions were compared with the baseline single-environment (SE) GS model (SE_CV1) representing a breeding scenario, where relationships and interactions are not leveraged across environments. Our results suggested that the ME models provide a clear advantage over SE models in terms of robust trait predictions. Both ME models provided 2–3 times higher prediction accuracies for all four traits across the four tested environments, highlighting the importance of accounting environmental variance in GS models. While the improvement in PA from SE to ME models was significant, the CV1 and CV2 schemes did not show any clear differences within ME, indicating the ME model was able to predict the untested environments and lines equally well. Overall, our results provide an important insight into the impact of environmental variation on GS in smaller breeding programs where these programs can potentially increase the rate of genetic gain by leveraging the ME wheat breeding trials.

## Introduction

Wheat (*Triticum aestivum* L.) is an essential cereal to secure global food security (Curtis and Halford, 2014). Significant efforts are needed to accelerate high-yielding varieties to fulfill future global wheat demand by 2050 (Hellin et al., 2012). Hence, the enhancement of grain yield (GRYLD) is a foremost target for wheat breeders. GRYLD is a complex trait administered by many small-effect loci with significant loci × loci interactions (Arzani and Ashraf, 2017; Sehgal et al., 2017). Moreover, the GRYLD trait is associated with strong genotype × environment interaction, which makes its genetic enhancement a difficult work.

Genomic selection (GS) integrates genome-wide dense markers and, as presented by Meuwissen et al. (2001), is a different marker-assisted selection approach. GS proves to be a powerful tool to improve the selection accuracy and prediction for quantitative traits in crop breeding (Crossa et al., 2017). GS utilizes a large set of, usually unidentified markers, spread over the whole genome in the same way as every quantitative trait locus (QTL) is in linkage disequilibrium (LD). GS is particularly beneficial for traits that cannot be evaluated on a few plants and for traits that are hard to estimate. It is still a vital issue for plant breeders to upsurge the accuracy of genomic prediction for selecting the advanced breeding lines.

The GS has been widely used in wheat breeding to predict various traits, such as yield, disease resistance, grain weight, heading, iron and zinc contents, end-use quality, and physiological traits (Charmet et al., 2014; Velu et al., 2016; Hayes et al., 2017; Juliana et al., 2017a,b; Norman et al., 2017; Lozada et al., 2019; Tomar et al., 2021). As such, GS embraces the prospects for the genomic enhancement of qualitative and quantitative traits. Many available GS models have been tested on various breeding and trait scenarios. Earlier numerous comparative studies of the GS model predictions in wheat showed that Random Forest and Reproducing Kernel Hilbert Space models performed better for traits of interest. However, any single GS model could not outperform other models (Pérez-Rodríguez et al., 2012; Charmet et al., 2014). Earlier studies have stated that many interconnected factors impact the overall model performance (Jannink et al., 2010; Heslot et al., 2012), such as heritability, population structure, statistical models, i.e., parametric and nonparametric models, cross-validation (CV) approaches, the genetics of traits, training population size and composition, marker density, and LD among markers and QTLs (Jannink et al., 2010; Pérez-Rodríguez et al., 2012; Crossa et al., 2017; Norman et al., 2018; Lozada et al., 2019).

The GS delivers the promise to accelerate genetic gain by increasing precision in selecting and shortening the breeding cycles. However, GS is a relatively new and advanced method for smaller and low-resource South Asian wheat breeding programs. Previously, substantial progress has been made in testing and validating various models for GRYLD and related traits in wheat in South Asia, albeit on larger breeding populations (De los Campos et al., 2009; Crossa et al., 2010, 2011, 2016; Heffner et al., 2011; Burgueño et al., 2012; Pérez-Rodríguez et al., 2012; Rutkoski et al., 2015; Juliana et al., 2017a,b, 2019; González-Camacho et al., 2018). These studies have highlighted the impact of environment and genotype × environment on the GS model performance. Therefore, to optimize the genetic gain from GS, the group of field-testing environments can be leveraged.

In this study, high-yielding, advanced wheat breeding lines from The International Maize and Wheat Improvement Center (CIMMYT) were evaluated for two consecutive wheat seasons (2017 and 2018) to adapt to the diverse environments of North and Central India. To evaluate the performance of multi-environment (ME) GS models on our specific set of selection environments, we tested different GS CV schemes mimicking the breeding schemes where untested lines and environmental performance are highly valuable to achieve the desired selection gains. This study is highly relevant particularly in the South Asian context where trial sizes are relatively small and broadly adapted wheat lines are sought after.

## Materials and Methods

### Plant Material

A set of 141 South Asian spring wheat lines (*T. aestivum* L.) were selected from the International Yield Trial of CIMMYT International Nurseries (elite germplasm). These lines constitute a diverse association panel. The seeds of 141 genotypes were obtained from the Germplasm Resource Unit, CIMMYT (Mexico). Detailed information with a pedigree for each genotype is given in Supplementary Table 1.

### Field Trials and Phenotypic Evaluation

The panel of selected lines was evaluated in field trials at the Borlaug Institute for South Asia (India) at Jabalpur (JBL) (23°14′00.6N and 80°04′40.7E) and Ludhiana (LDH) (30°59′28.74N and 75°44′10.87E), locations during the crop season for 2 years (2017 and 2018), genotypes were phenotyped and evaluated across all trials for four traits [days to maturity (DAYSMT), days to heading (DTHD), GRYLD, and thousand-grain weight (TGW)] (Supplementary Table 2). The experiment was conducted in two replications following the Randomized Block Design (RBD). The normal agronomic practice was followed for trial management. The row-to-row distance was maintained at 20 cm.

### Genotyping-by-Sequencing and SNP Filtering

Genomic DNA was extracted from the fresh leaves of seedling wheat using the modified cetyltrimethylammonium bromide (CTAB) method (Dreisigacker et al., 2016). Genotyping-by-sequencing (GBS) was performed in Illumina HiSeq 2500 using a protocol suggested by Poland et al. (2012). Single nucleotide polymorphism (SNP) calling was performed using TASSEL version 5.2.43 (Bradbury et al., 2007) using the TASSEL-GBSv2 pipeline. Using Beagle version 4.1, the missing data were imputed with default settings. After quality control (filter criteria: sample call rate > 0.8, Minor allele frequency (MAF) ≥ 0.05, SNP call rate > 0.7), 14,563 polymorphic SNPs and 141 genotypes were selected for the follow-up analysis (Supplementary Table 3). Among polymorphic SNP markers, 40.66, 50.66, and 8.68% were from the A, B, and D genomes, respectively. With a genomic coverage of 13.9 GB and 14,563 markers across the genome, the average marker density was one marker per 0.95 Mb. The highest marker density with one marker per 0.54 Mb of chromosome 2B and the lowest marker density with one marker per 6.854 Mb at chromosome 4D were observed.

### Statistical Analysis of Phenotypes

Each location-year combination is treated as a distinct environment for analysis purposes. Broad-sense heritability for each trait/environment combination was estimated at the plot level, and raw phenotypic values were adjusted to derive the best linear unbiased predictions (BLUPs) (Supplementary Table 4) for each trait at each environment using META-R (Alvarado et al., 2020) by using the following formula:


$$\begin{array}{l} Y_{ik}=\mu +Rep_{i}\;+\;Gen_{k}\;+\epsilon_{ik}(\text{within}\;\text{environments})\\ Y_{ijk}=\mu +Env_{i}\;+\;Rep_{j}\left(Env_{i}\right)\;+Gen_{k}+Env_{i}\times Gen_{k}\\ \;+\epsilon_{ijk}(\text{across}\;\text{environments}) \end{array}$$

where *Y*_*ik*_ is the trait of interest, μ is the mean effect, *Rep*_*i*_ is the effect of the *i*th replicate, *Gen*_*k*_ is the effect of the *k*th genotype, ϵ_*ik*_is the error associated with the *i*th replication and the *k*th genotype, which is assumed to be normally and independently distributed, with mean 0 and homoscedastic variance. For across environments, *Y*_*ijk*_ is the trait response and the *i*th environment, *Rep*_*j*_(*Env*_*i*_) is the effect of *j*th Rep in the *i*th environment, and *Env*_*i*_×*Gen*_*k*_ is the environment × genotype interaction. The resulting analysis produced the adjusted trait phenotypic values in the form of BLUPs within and across environments. The BLUPs model considers genotypes as random effects, minimizing the effect of screening time and other environmental effects.

In addition, the components of the phenotypic variance of a given trait at an individual environment and across environments were also extracted to calculate the broad-sense heritability using the formula as follows:


$$\begin{array}{l} H^{2}=\frac{\sigma_{g}^{2}}{\sigma_{g}^{2}\;+\;\frac{\sigma_{e}^{2}}{nReps}}(\text{within}\;\text{environments})\\ H^{2}=\frac{\sigma_{g}^{2}}{\sigma_{g}^{2}\;+\;\frac{\sigma_{ge}^{2}}{nEnvs}\;+\;\frac{\sigma_{e}^{2}}{\left(nEnvs\;\times \;nReps\right)}}\;(\text{across}\;\text{environments}) \end{array}$$

where $\sigma_{g}^{2}\;$and $\sigma_{e}^{2}$ are the genotype and error variance components, respectively, $\sigma_{ge}^{2}$ is genotype × environment interaction variance, nEnvs is the number of environments, and nReps is the number of replicates. All effects are considered random for calculating the BLUPs (Supplementary Table 4) and the broad-sense heritability. The BLUPs phenotypic distributions of GRYLD and other traits at each environment were plotted to check normality assumptions. Phenotypic and genetic correlations were calculated for each trait and environment combination in R software version 4.0.2. (R Core Team, 2019) using FactoMineR version 2.4 (Lê et al., 2008) and factoextra version 1.0.7 (Kassambara and Mundt, 2020).

### Baseline Single-Environment (SE) Genomic BLUP Model (GBLUP), CV Schemes, and Predictive Ability

The baseline SE genomic prediction analysis was implemented in the BWGS program (Charmet et al., 2020). BWGS performs a GBLUP analysis using a marker-based relationship matrix. CV delivers an unbiased evaluation for the performance of a GS model; therefore, a 5-fold CV approach was implemented for reducing the unwanted bias (Kohavi, 1995), where the genotypes (for each trait separately) were randomly split into five equal-sized folds. SE_CV1 model was fitted with missing phenotypic values for the tested individuals. Prediction accuracy (PA) was subsequently calculated as the correlation of predicted breeding values with the observed phenotypic values for the missing genotypes. This step was repeated for each environment and fold separately. The genomic PA was then calculated by iteratively assigning 1-fold as the validation set and the remaining folds as the training set. This five-fold validation process was repeated 50 times to randomly shuffle the lines in each fold. The accuracy of the genomic predictions was measured as the Pearson's correlation between the predicted and actual trait BLUPs.

A mixed model of the simplified form was fitted for genomic predictions as follows:


$$\begin{array}{l} \text{y}=X\text{b}+Z\text{g}+\text{e} \end{array}$$

where *y* is a vector of adjusted phenotypes, X is a design matrix relating the fixed effects to each genotype, *b* is a vector of fixed effects, Z is a design matrix connecting records to genetic values, *g* is a vector of additive genetic effects for a genotype, and *e* is a vector of random normal deviates with variance $\delta_{e}^{2}$.

### Advanced ME GBLUP Model, CV Schemes, and Predictive Ability

**Figure 1:**
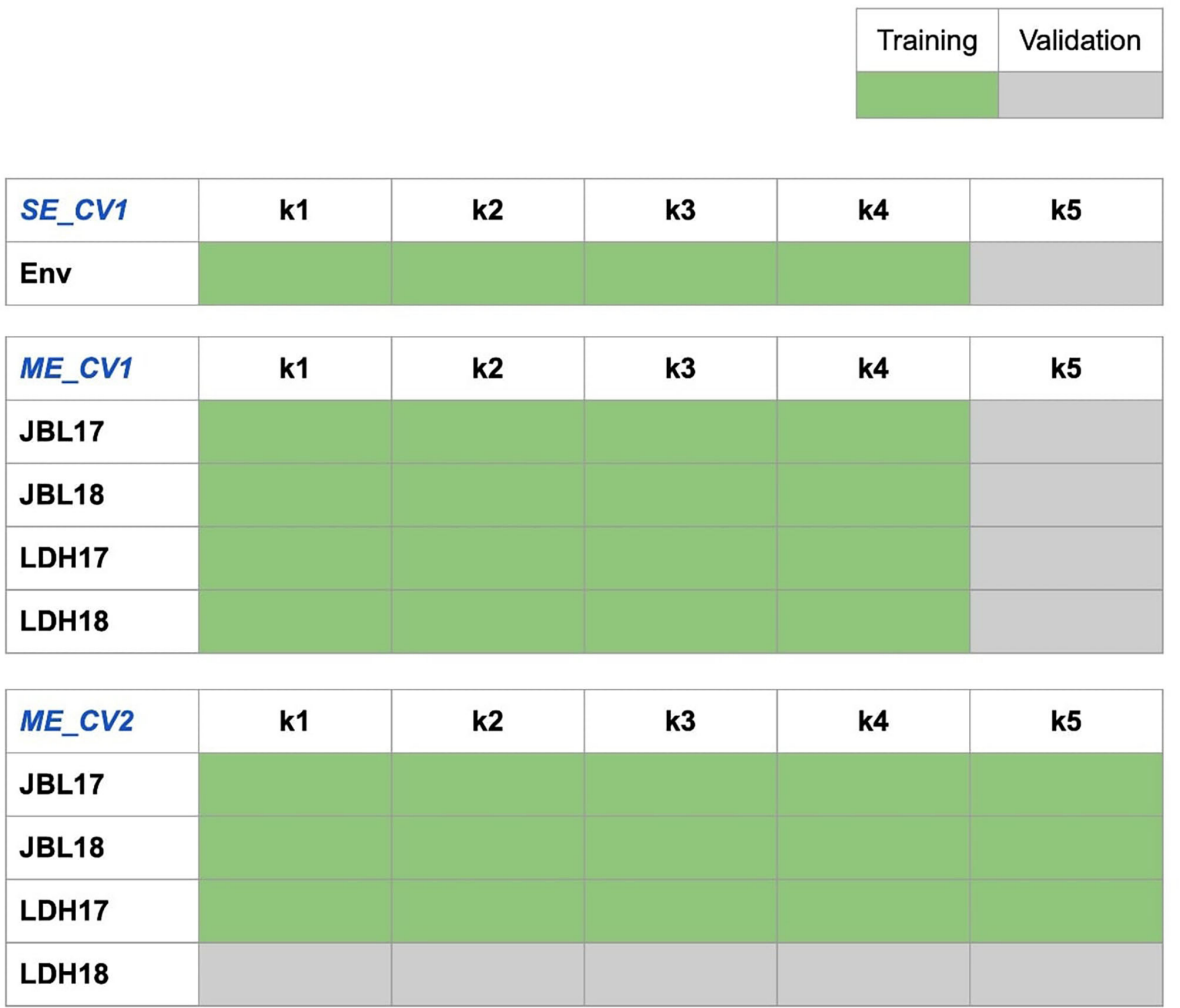
Prediction scheme for the single-environment (SE) and multi-environment (ME) genomic prediction models with two cross-validation schemes (CV1 and CV2) used in this study. SE_CV1 model: the SE prediction model with CV scheme 1 where a trait [e.g., grain yield (GRYLD)] is predicted at a time; we used 80% of individuals as the training set (phenotyped and genotyped, light green) and 20% of the individuals as the testing set (genotyped only, light gray with validation code for the trait to be predicted, yield as an example here). ME_CV1 model: the ME prediction model with CV scheme 1 for new un-phenotyped individuals; we used 80% of individuals as the training set (phenotyped for all traits and genotyped; light green) and 20% of the individuals as the validation set (genotyped but not phenotyped for any trait; light gray with validation code for the trait to be predicted, GRYLD as an example here). ME_CV2 model: the ME prediction model with CV scheme 2 where 100% of the information from other traits are available for the individuals to be predicted; we used 80% of individuals as the training set (phenotyped for all traits and genotyped; light green) and 20% of individuals as the validation set (phenotyped for associated traits but not for the targeted traits, and genotyped; light gray with predication code for the trait to be predicted, yield as an example here). Rectangles represent genotypes, and colors represent whether the phenotypic information was used (light green) or not (light gray with validation code for the trait to be predicted, GRYLD as an example) for the population. A similar scheme was applied for predicting days to heading (DTHD), days to maturity (DAYSMT), and thousand-grain weight (TGW).

The advanced ME genomic prediction analysis was implemented in Solving Mixed Model Equations in the R (sommer) package (Covarrubias-Pazaran, 2016). Two types of ME_CV schemes representing actual breeding scenarios were implemented. The first scenario represents a use case where some genotypes are missing across all environments (ME_CV1). ME_CV1 was fitted by masking the phenotypic values of genotypes belonging to the test set across all environments. PA was calculated as the correlation of predicted and observed phenotypic values for the missing genotypes at each environment separately. In the second scenario, the entire trial or all genotypes are missing at one of the environments (ME_CV2). ME_CV2 was fitted by masking the phenotypic values of all lines in an SE. The trained model was then used to predict the breeding values of lines in the missing environment. PA was calculated as the correlation of predicted and observed phenotypic values of the tested lines. The CV schemes are illustrated in Figure 1.

In ME genomic predictions, the SE model was rewritten and implemented as follows:


$$\begin{array}{l} y_{ij}=g_{j}+E_{i}+gE_{ij}+e_{ij} \end{array}$$

where *y*_*ij*_ represents response of *j*th line in the *i*th environment (*i* = 1, 2,……*i, j* = 1, 2,…… *j*; *g*_*j*_ is the effect of *j*th line with *g* = (*g*_1…….._*g*_*j*_)T~N(0, $\delta_{1}^{2}$G*g*), $\delta_{1}^{2}$ is the genomic variance, G*g* is the genomic relationship matrix. *E*_*i*_ represents the effect of the *i*th environment. *gE*_*ij*_ is the random term that takes into account the interaction between the genomic effect of *j*th line and the *i*th environment with *gE*= (*g*_1_………*g*_*j*_)T~N (0, $\delta_{2}^{2}I_{I}⊗$ G), where $\delta_{2}^{2}\;$is the interaction variance. *Eij* is a random residual effect of the *j*th line in the *i*th environment [N (0, $\delta_{2}^{2}$)], where $\delta_{2}^{2}$ is the residual variance.

## Results

### Heritability, Correlations, and Trait Characterization

A range of variation was detected for GRYLD and other related traits across environments/years (LDH17 and LDH18 and JBL17 and JBL18). The highest averaged GRYLD over environments/years was observed at JBL18 (9.4 ton/ha), followed by JBL17 (8.7 ton/ha), LDH17 (8.2 ton/ha), and LDH18 (7.9 ton/ha). Similarly, the TGW trait also showed variation across environments. The highest averaged TGW over environments/years was observed at JBL17 (69 g), followed by JBL18 (59.5 g), LDH17 (58.4 g), and LDH18 (53.5 g). We observed significant G × E interaction for the GRYLD and DAYSMT in JBL18 and LDH17 (Tables 1, 2). For all traits, the broad-sense heritability ranged from 0.47 to 0.96. The broad-sense heritability of DTHD was the highest (0.96) in LDH17, while GRYLD, the lowest (0.47) was in JBL18, and the highest (0.74) was in LDH17. TGW had the highest stability and relatively high heritability (0.80–0.86) across environments.

**Table 1:** Variability analysis of various yield-related agronomic traits for four environments at two locations.

**Loc^#^**	**Env**	**Trait^##^**	***H* ^**2**^**	**G Var**	**R Var**	**G Mean**	**LSD**	**CV**	**G Sig**
JBL	JBL17	DTHD	0.84	10.98	4.04	81.96	3.65	2.45	0
		DAYSMT	0.86	5.86	1.89	124.82	2.52	1.10	0
		GRYLD	0.48	0.29	0.63	7.87	1.08	10.09	0.000151
		TGW	0.86	26.59	8.92	54.66	5.47	5.47	0
	JBL18	DTHD	0.78	12.79	7.30	79.26	4.71	3.41	0
		DAYSMT	0.71	4.89	3.96	124.67	3.32	1.60	9.08E-13
		GRYLD	0.47	0.15	0.34	8.76	0.79	6.67	0.000172
		TGW	0.80	12.53	6.34	46.22	4.45	5.45	0
LDH	LDH17	DTHD	0.96	12.61	1.19	94.85	2.11	1.15	0
		DAYSMT	0.74	4.79	3.29	148.73	3.09	1.22	6.88E-15
		GRYLD	0.74	0.21	0.15	7.06	0.66	5.55	2.73E-14
		TGW	0.81	15.42	7.03	45.48	4.73	5.83	0
	LDH18	DTHD	0.88	8.58	2.44	103.71	2.89	1.51	0
		DAYSMT	0.88	8.18	2.25	144.52	2.80	1.04	0
		GRYLD	0.62	0.16	0.20	7.26	0.69	6.11	1.92E-08
		TGW	0.83	14.66	6.13	44.30	4.47	5.59	0

# *Loc, location; Env, Environment; H^2^, heritability; G Var, genotypic variance; R Var, residual variance; LSD, least significant difference; CV, critical variance; G Sig, genotypic significance; LDH, Ludhiana; JBL, Jabalpur*.

## *DTHD, days to heading; DAYSMT, days to maturity; GRYLD, grain yield; TGW, thousand-grain weight*.

**Table 2:** Variability analysis of various yield-related agronomic traits for four environments at two locations.

**Traits**	***H* ^**2**^**	**G Var**	**G × E Var**	**R Var**	**G Mean**	**LSD**	**CV**	**n Rep**	**n Env**	**G Sig**	**G × E Sig**
DTHD	0.90	8.94	2.29	3.74	89.94	2.69	2.15	2	4	8.93E-73	1.16E-18
DAYSMT	0.83	4.00	1.94	2.83	135.68	2.32	1.24	2	4	4.34E-44	2.01E-21
GRYLD	0.38	0.05	0.15	0.33	7.74	0.49	7.43	2	4	0.0003	3.69E-13
TGW	0.78	9.90	7.41	7.10	47.67	4.07	5.59	2	4	1.13E-33	4.23E-35

*H^2^, heritability; G Var, genotypic variance; R Var, residual variance; LSD, least significant difference; CV, critical variance; G Sig, genotypic significance; DTHD, days to heading; DAYSMT, days to maturity; GRYLD, grain yield; TGW, thousand-grain weight*.

**Figure 2:**
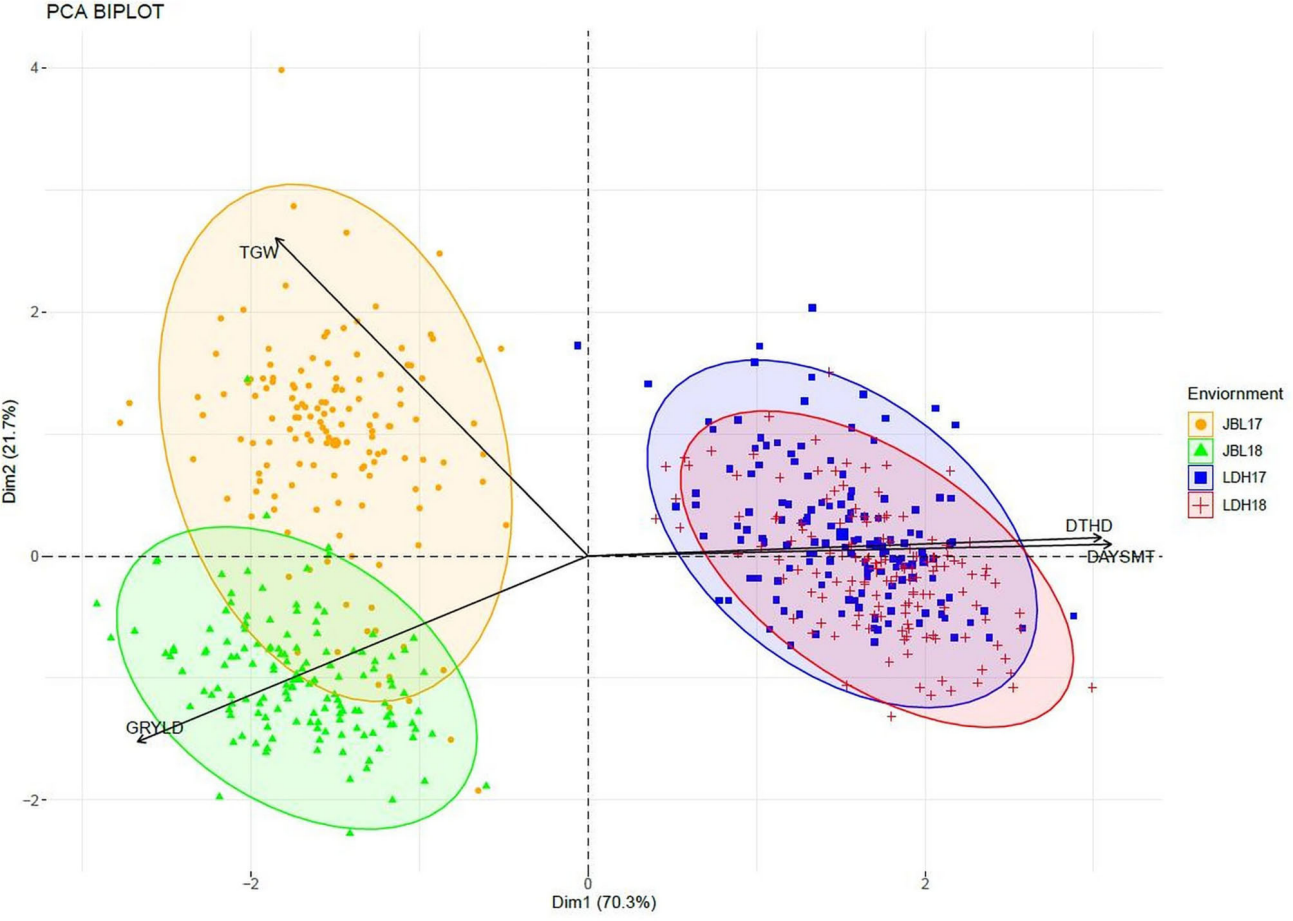
The principal component analysis shows the correlation among GRYLD, TGW, DAYSMT, and DTHD in four environments (LDH17, LDH18, JBL17, and JBL18).

The phenological traits DTHD and DAYSMT displayed the strongest positive correlation (0.88), followed by a weak positive correlation TGW-GRYLD (0.15), while GRYLD and DTHD (−0.73) demonstrated negative correlations. The lowest correlation was observed between GRYLD and DAYSMT (−0.76) traits. The principal component analysis (PCA) of multivariate analysis enables the easier understanding of effects and networks among different traits and elucidates genotypic difference among a set of given traits, i.e., the first two PCs explained 92% of the total variation. The PC1 explained 70.3% of the total variance and PC2 explained 21.7% of the total variance (Figure 2).

### Baseline SE Model: Performance of Untested Lines in the Same Environment

**Figure 3:**
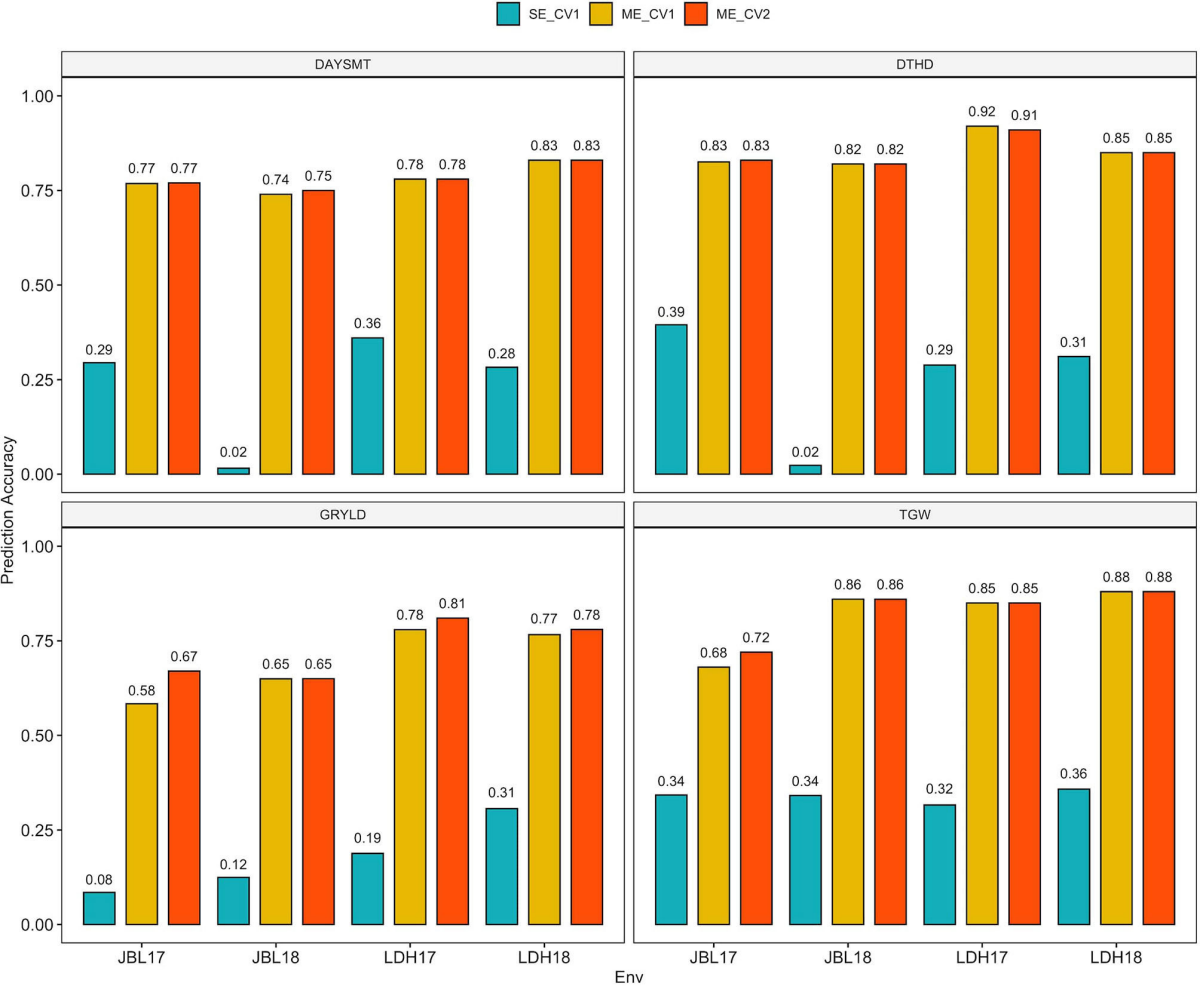
Bar plots showing the prediction accuracy (PA) of DAYSMT, DTHD, GRYLD, and TGW using SE and ME models from individual experiments across locations (LDH17, LDH18, JBL17, and JBL18). SE_CV1 predicting SE at a time, ME_CV1 predicting new lines with genotypic information only, and ME_CV2 predicting partially phenotyped lines by using genotypic and phenotypic information from all traits from individuals in the training set, and genotypic and correlated phenotypic traits in the testing set.

A GS scenario representing SE breeding programs was tested. In this model, the PAs of the GS models for each of the four traits were separately generated for all four tested environments. In other words, the environments were treated as independent. Overall, the PA of the SE model was significantly lower among the three tested GS scenarios (**Table 4**; Figure 3). PA was the highest for TGW (0.34) and the lowest for GRYLD (0.18) traits. A relatively low moderate PA ranging between 0.24 and 0.25 was observed for DAYSMT and DTHD traits. Among the tested environments, JBL18 had the lowest overall PA (0.01–0.02) compared to the rest of the three environments for DTHD and DAYSMT (0.25–0.40). TGW was the only trait where a highly consistent and moderate PA (0.32–0.35) across all environments was observed. PA for GRYLD was the highest for LDH18 (0.32) and the lowest for JBL17 (0.08).

### Advanced ME Model: Performance of Tested Lines in Untested Environments and Untested Lines in Tested Environments

The inclusion of environmental information in ME models significantly improved the PA over SE models across all traits and environments (Figure 3). A very high and consistent PA ranging from 0.69 to 0.85 was observed for all traits and environments for both ME models (ME_CV1 and ME_CV2). The most considerable improvement in PA due to ME was observed for the GRYLD trait, where PA increased from 0.18 to 0.73 for SE and ME models (**Table 4**). Interestingly, identical trait rankings were also observed for two ME models, where the DTHD ranked the highest (0.85) and GRYLD ranked the lowest (0.69–0.73) among all four traits. While the ME models had identical trait rankings, the environments ranked slightly differently for the two models for all traits. For instance, both years (2017 and 2018) at the LDH location had higher overall PA compared to JBL for all traits.

## Discussion

Crop breeders regularly evaluate the performance of genotypes and collect multiple traits data in various environments. The genotype-based selection on phenotypic and GBS marker information using genomic prediction models is gradually acquiring acceptance in breeding with the initiation of economical next-generation sequencing (NGS) technologies (Poland and Rife, 2012). Limited study has been conducted using the multi-environment genomic prediction (ME-GP) methods due to the complexity and higher computing requirements (Oakey et al., 2016; Rincent et al., 2017; Montesinos-López et al., 2018; Roorkiwal et al., 2018; Bhandari et al., 2019; Tolhurst et al., 2019; Pandey et al., 2020).

### Trait Correlation and Characterization: A Vital Factor for Improving Accuracy in ME-GP

**Figure 4:**
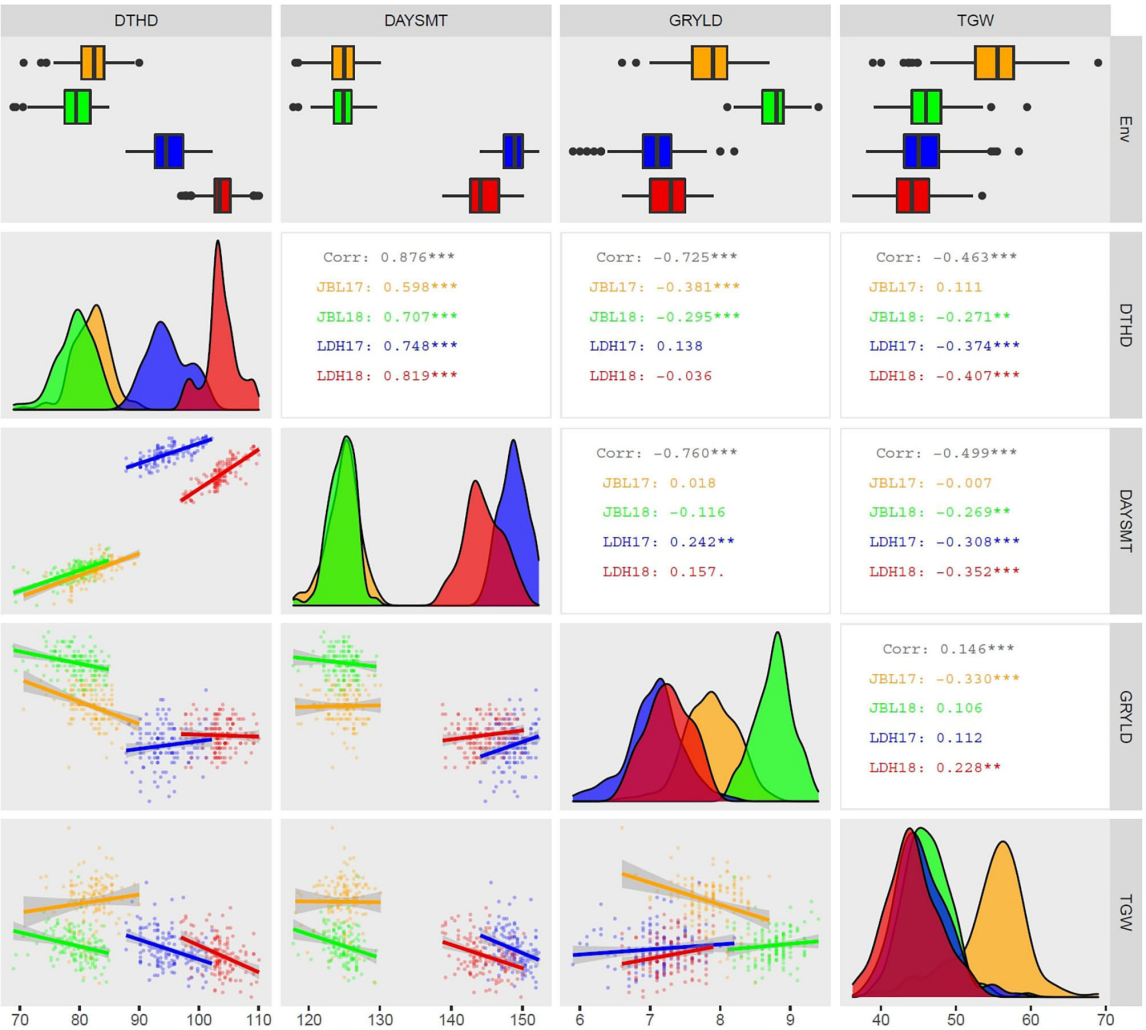
Distributions, scatter plots, and correlations between agronomic traits using best linear unbiased predictions from combining and four experiments [Ludhiana (LDH)17, LDH18, Jabalpur (JBL)17, and JBL18]. The distribution of DTHD, DAYSMT, GRYLD, and TGW values is displayed on the diagonal with environments indicated by colors. The top row represents the distribution of traits as boxplots. The upper right triangle shows pairwise correlation values as overall correlation in black color while other colors are represented individually as explained earlier. The correlations among environments are displayed as scatter plots in the lower triangular area and as the Pearson's correlation coefficients in the upper triangular area. Numbers indicate a correlation that is significantly different from 0 at an alpha level of 0.05. DTHD, DAYSM, GRYLD, and TGW. ## level of significance; ***p < 0.001, **p < 0.01, *p < 0.1, and p < 0.15.

In this study, advanced breeding lines as part of the bread wheat program of CIMMYT were evaluated under irrigated conditions at two locations (JBL and LDH) for 2 years (2017 and 2018) (i.e., four environments). This study evaluated four traits (i.e., DTHD, DAYSMT, GRYLD, and TGW) for use in an ME trait GP model. GRYLD and related traits were positively correlated to each other in two sets (i.e., 1: DAYSMT and DTHD; and 2: GRYLD and TGW) (Figure 4). This positive correlation of GRYLD with TGW in this study points out that the GRYLD was mainly distinct by the TGW factor. The negative relationship between GRYLD and DTHD indicates that the early-headed genotypes play a vital role in the stability of advanced breeding line yield during grain filling and finally affecting the yield component (Sharma and Smith, 1986).

### Yield and Related Trait Heritability Difference Among Environments

Our results showed that the heritability of the traits ranged from moderate (i.e., GRYLD) to high (i.e., DAYSMT, DTHD, and TGW). Among the four traits, the phenological traits (i.e., DTHD and DAYSMT) and TGW particularly showed high stable broad-sense heritability ranging from 0.71 to 0.96. It suggests the high quality of the phenotypic measurements and significant predictive potential of the traits. GRYLD, a highly quantitative and environmentally sensitive trait (Maphosa et al., 2014; Würschum et al., 2018), showed considerable fluctuation across environments with JBL environment having relatively lower heritability (0.47–0.48) compared to LDH (0.62–0.74). The variance explained by agronomic traits was significant (Table 1) and indicating a large G × E impact on GRYLD resulted in a lower heritability compared to other traits. Hence, lower heritability estimates for GRYLD were expected as numerous genes govern it. The low heritability and yield variances also could be the possible effect of the smaller plot size and lower sowing density (Rode et al., 2011; Sallam et al., 2015; Thorwarth et al., 2017; Bhatta et al., 2018) (Tables 1, 2). The climate in these two environments is considerably different. While the growing season length is relatively shorter in JBL due to the high overall temperature, the LDH location has a moderately colder climate and longer growing season (Mondal et al., 2016). On the one hand, these highly variable environments do underscore a highly challenging phenotypic landscape; it also presents a significant opportunity to leverage the ME trial framework for trait improvement (Lillemo et al., 2005; Braun et al., 2010). The presence of significant genetic and environmental correlations (i.e., positive correlation in TGW and GRYLD, and DAYSMT and DTHD) in our experiments led us to hypothesize that the correlated traits and environmental relationships can be leveraged to improve the selection accuracy through marker-based ME-GS models (Figure 4). Therefore, we proceeded with applying the ME model to test this hypothesis on our selected set of lines (Table 3).

**Table 3:** Genetic and phenotypic correlations in agronomic important traits.

	**Genetic correlations**				**Phenotypic correlations**		
**Traits**	**DTHD**	**DAYSMT**	**GRYLD**	**Traits**	**DTHD**	**DAYSMT**	**GRYLD**
DAYSMT	0.94			DAYSMT	0.83		
GRYLD	−0.30	−0.29		GRYLD	−0.22	−0.08	
TGW	−0.33	−0.26	0.22	TGW	−0.29	−0.24	0.07

*DTHD, days to heading; DAYSMT, days to maturity; GRYLD, grain yield; TGW, thousand-grain weight*.

### SE and ME Genomic Prediction Across Years and Sites and ME Model Utilities in Crop Breeding

While weak predictive capability continues to be a major issue in successfully applying GS (Crossa et al., 2013), numerous studies have demonstrated that GS could be beneficial for quantitative traits such as GRYLD with low heritability and also on how GS can be utilized in a breeding program by using even low to moderate GP in early generation selection (Belamkar et al., 2018; Lado et al., 2018; Michel et al., 2018). There are several aspects influencing the PA of GP models. Some of the crucial aspects associated with this study of ME were the genetic relationship between the testing and training sets, the size of the training set, heritability and trait architecture, and correlations among traits and environments (Asoro et al., 2011; Crossa et al., 2013; Heslot et al., 2013; Sallam et al., 2015; Zhang et al., 2015; Duangjit et al., 2016; Lado et al., 2016; Wang et al., 2016; Thorwarth et al., 2017; Akdemir and Isidro-Sánchez, 2019; Olatoye et al., 2020). Even though the size of the population was small in our study, the GP using correlated traits in the ME_CV1 and ME_CV2 schemes had higher PA, indicating that correlated traits up to some extent could balance the impact on the sizes of small population.

Models that leverage E and G × E components have been shown to improve the genomic prediction accuracies for highly quantitative traits such as phenology and GRYLD (Burgueño et al., 2012; Dias et al., 2018). To evaluate the potential of genomic predictions in highly productive but variable environments of JBL and LDH, we simulated three different genomic prediction scenarios representing actual breeding programs. A comparison of single and ME models showed a 2- to 3-fold improvement in model performance for all traits (Table 4; Figure 3). Among the four traits, GRYLD showed the highest (3.8X) absolute increase in PA from SE to ME models, highlighting the significance of ME modeling in GRYLD predictions. For the SE model, TGW had the most consistent PA across four environments (0.32–0.34), which was in agreement with the highly stable heritability and a lower fraction of G × E observed for this trait (Table 2; Figure 3). Interestingly, the PA of the two ME models (CV1 and CV2) showed no significant change, suggesting that the ME model was able to predict well the untested environments and lines equally. A model can be highly predictive of untested environments in scenarios where environments are highly correlated (Malosetti et al., 2016; Jarquín et al., 2017), which seems to be the case for our environments as reflected by the low G × E and high heritability (Table 1; Figure 3). Similarly, a remarkable improvement in the predictive performance of ME_CV1 can be partially attributed to the fact that our sampled set of lines came from the same breeding program and the sample size of 141 lines was relatively moderate. From the perspective of a breeding program, the strong performance of the two ME models suggests that our breeding program can increase the overall population size without losing any significant predictive power through sparse testing at these two environments (Cullis et al., 2020; Jarquin et al., 2020). A high population size from the sparse testing framework here can deliver a high selection gain through increased selection intensity.

**Table 4:** Genomic prediction accuracies averaged across four environments for four traits and three modeling scenarios (a) single-environment CV1 (SE_CV1), (b) multi-environment CV1 (ME_CV1), and (c) multi-environment CV2 (ME_CV2).

**Traits**	**Average prediction accuracy**		
	**SE_CV1**	**ME_CV1**	**ME_CV2**
DAYSMT	0.24	0.78	0.78
DTHD	0.25	0.85	0.85
GRYLD	0.18	0.69	0.73
TGW	0.34	0.82	0.83

*DTHD, days to heading; DAYSMT, days to maturity; GRYLD, grain yield; TGW, thousand-grain weight*.

## Conclusion

Breeding for quantitative traits is challenging due to the complex genetic architecture of traits that are highly affected by the complex G × E interactions in field trials. A suitable genomic prediction modeling strategy can potentially address this challenge through ME genomic prediction models. In this study, we evaluated genomic prediction accuracies of advanced spring wheat lines under four diverse environments in two wheat-growing regions in India. The ME-GS models showed significant improvement over SE models in terms of prediction accuracies. Our results suggest that ME can be leveraged to improve the breeding selection efficiency for major agronomic and phonological traits. Over the years, CIMMYT has established an extensive network of field-testing sites in South Asian countries including India, Pakistan, Bangladesh, and Nepal. Our results suggest that the wheat breeding programs in these countries can greatly benefit from GS through better modeling of environmental variance and sparse testing of a larger cohort of breeding lines. Future research efforts will be directed toward including high-throughput phenotyping traits such as plant height, Normalized Difference Vegetation Index (NDVI), and senescence into the genomic prediction framework to improve the selection efficiency of spring wheat in the South Asian breeding programs.

## Data Availability Statement

The datasets presented in this study can be found in online repositories. The names of the repository/repositories and accession number(s) can be found in the article/Supplementary Material.

## Author Contributions

VT and DS drafted the manuscript. VT, DS, GD, and YC analyzed the data. UK, JP, and RS designed the field trials, conducted genotyping, and provided breeding lines. VT and YG collected field data. UK, BT, JP, RS, and AJ supervised the overall study. All authors contributed to the article and approved the submitted version.

## Funding

This study was supported by the United States Agency for International Development (USAID), Feed the Future Innovation Lab for Applied Wheat Genomics (Cooperative Agreement No. AID-OAA-A-13-00051), and CGIAR Research Program on Wheat (CRP) Partner Grant to BISA (Grant Code: A5017.09.64).

## Conflict of Interest

The authors declare that the research was conducted in the absence of any commercial or financial relationships that could be construed as a potential conflict of interest.

## Publisher's Note

All claims expressed in this article are solely those of the authors and do not necessarily represent those of their affiliated organizations, or those of the publisher, the editors and the reviewers. Any product that may be evaluated in this article, or claim that may be made by its manufacturer, is not guaranteed or endorsed by the publisher.

## Supplementary Material

Supplementary Figure 1 Weather information of LDH17 and LDH18.

Supplementary Figure 2 Weather information of JBL17 and JBL18.

Supplementary Table 1 List of 141 genotypes with pedigree information used in this study.

Supplementary Table 2 List of traits that were evaluated during this study in the field trials.

Supplementary Table 3 GBS HapMap data used in this study.

Supplementary Table 4 Best linear unbiased predictions (BLUPs) data used in this study.

## References

[B1] Akdemir D. Isidro-Sánchez J. (2019). Design of training populations for selective phenotyping in genomic prediction. Sci. Rep. 9, 1–15. 10.1038/s41598-018-38081-6 30723226 PMC6363789

[B2] Alvarado G. Rodríguez F. M. Pacheco A. Burgueño J. Crossa J. Vargas M.. (2020). META-R: a software to analyze data from multi-environment plant breeding trials. Crop J. 8, 745–756. 10.1016/j.cj.2020.03.010

[B3] Arzani A. Ashraf M. (2017). Cultivated ancient wheats (*triticum spp*.): a potential source of health-beneficial food products. Compr. Rev. Food Sci. Food Saf. 16, 477–488. 10.1111/1541-4337.12262 33371554

[B4] Asoro F. G. Newell M. A. Beavis W. D. Scott M. P. Jannink J. (2011). Accuracy and training population design for genomic selection on quantitative traits in elite north american oats. Plant Genom. 4:007. 10.3835/plantgenome2011.02.0007

[B5] Belamkar V. Guttieri M. J. Hussain W. Jarquín D. El-basyoni I. Poland J.. (2018). Genomic selection in preliminary yield trials in a winter wheat breeding program. G3 Genes, Genomes, Genet. 8, 2735–2747. 10.1534/g3.118.200415 29945967 PMC6071594

[B6] Bhandari A. Bartholom,é J. Cao-Hamadoun T.-V. Kumari N. Frouin J. Kumar A.. (2019). Selection of trait-specific markers and multi-environment models improve genomic predictive ability in rice. PLoS ONE 14:e0208871. 10.1371/journal.pone.0208871 31059529 PMC6502484

[B7] Bhatta M. Morgounov A. Belamkar V. Baenziger P. S. (2018). Genome-Wide association study reveals novel genomic regions for grain yield and yield-related traits in drought-stressed synthetic hexaploid wheat. Int. J. Mol. Sci. 19:3011. 10.3390/ijms19103011 30279375 PMC6212811

[B8] Bradbury P. J. Zhang Z. Kroon D. E. Casstevens T. M. Ramdoss Y. Buckler E. S. (2007). TASSEL: software for association mapping of complex traits in diverse samples. Bioinformatics 23, 2633–2635. 10.1093/bioinformatics/btm308 17586829

[B9] Braun H. J. Atlin G. Payne T. (2010). Multi-location testing as a tool to identify plant response to global climate change, in Climate Change and Crop Production, Reynolds M. P.. (CABI International), 115–138.

[B10] Burgueño J. Campos G. de los Weigel K. Crossa J. (2012). Genomic prediction of breeding values when modeling genotype × environment interaction using pedigree and dense molecular markers. Crop Sci. 52, 707–719. 10.2135/cropsci2011.06.0299

[B11] Charmet G. Storlie E. Oury F. X. Laurent V. Beghin D. Chevarin L.. (2014). Genome-wide prediction of three important traits in bread wheat. Mol. Breed. 34, 1843–1852. 10.1007/s11032-014-0143-y 26316839 PMC4544631

[B12] Charmet G. Tran L.-G. Auzanneau J. Rincent R. Bouchet S. (2020). BWGS: A R package for genomic selection and its application to a wheat breeding programme. PLoS ONE 15:e0222733. 10.1371/journal.pone.0222733 32240182 PMC7141418

[B13] Covarrubias-Pazaran G. (2016). Genome-Assisted prediction of quantitative traits using the R package sommer. PLoS ONE 11:e0156744. 10.1371/journal.pone.0156744 27271781 PMC4894563

[B14] Crossa J. Campos G. de los Pérez P. Gianola D. Burgueño J. Araus J. L.. (2010). Prediction of genetic values of quantitative traits in plant breeding using pedigree and molecular markers. Genetics 186, 713–724. 10.1534/genetics.110.118521 20813882 PMC2954475

[B15] Crossa J. Jarquín D. Franco J. Pérez-Rodríguez P. Burgueño J. Saint-Pierre C.. (2016). Genomic prediction of gene bank wheat landraces. G3 Genes|Genomes|Genetics 6:1819. 10.1534/g3.116.029637 27172218 PMC4938637

[B16] Crossa J. Pérez P. Campos G. de los Mahuku G. Dreisigacker S. Magorokosho C. (2011). Genomic selection and prediction in plant breeding. J. Crop Improv. 25, 239–261. 10.1080/15427528.2011.55876733970915

[B17] Crossa J. Pérez P. Hickey J. Burgueño J. Ornella L. Cerón-Rojas J.. (2013). Genomic prediction in CIMMYT maize and wheat breeding programs. Hered 112, 48–60. 10.1038/hdy.2013.16 23572121 PMC3860161

[B18] Crossa J. Pérez-Rodríguez P. Cuevas J. Montesinos-López O. Jarquín D. de los Campos G.. (2017). Genomic selection in plant breeding: methods, models, and perspectives. Trends Plant Sci. 22, 961–975. 10.1016/j.tplants.2017.08.011 28965742

[B19] Cullis B. R. Smith A. B. Cocks N. A. Butler D. G. (2020). The design of early-stage plant breeding trials using genetic relatedness. J. Agric. Biol. Environ. Stat. 25, 553–578. 10.1007/s13253-020-00403-5

[B20] Curtis T. Halford N. G. (2014). Food security: the challenge of increasing wheat yield and the importance of not compromising food safety. Ann. Appl. Biol. 164, 354–372. 10.1111/aab.12108 25540461 PMC4240735

[B21] De los Campos G. Naya H. Gianola D. Crossa J. Legarra A. Manfredi E.. (2009). Predicting quantitative traits with regression models for dense molecular markers and pedigree. Genetics 182, 375–385. 10.1534/genetics.109.101501 19293140 PMC2674834

[B22] Dias K. O. D. G. Gezan S. A. Guimarães C. T. Nazarian A. da Costa e Silva L. Parentoni S. N.. (2018). Improving accuracies of genomic predictions for drought tolerance in maize by joint modeling of additive and dominance effects in multi-environment trials. Hered 121, 24–37. 10.1038/s41437-018-0053-6 29472694 PMC5997769

[B23] Dreisigacker S. Deepmala S. Jaimez R. A. Luna-Garrid B. Muñoz-Zavala S. Núñez-Ríos C.. (2016). CIMMYT Wheat Molecular Genetics: Laboratory Protocols and Applications to Wheat Breeding. Mexico, DF: CIMMYT.

[B24] Duangjit J. Causse M. Sauvage C. (2016). Efficiency of genomic selection for tomato fruit quality. Mol. Breed. 36, 1–16. 10.1007/s11032-016-0453-3

[B25] González-Camacho J. M. Ornella L. Pérez-Rodríguez P. Gianola D. Dreisigacker S. Crossa J. (2018). Applications of machine learning methods to genomic selection in breeding wheat for rust resistance. Plant Genome 11:170104. 10.3835/plantgenome2017.11.0104 30025028 PMC12962436

[B26] Hayes B. J. Panozzo J. Walker C. K. Choy A. L. Kant S. Wong D.. (2017). Accelerating wheat breeding for end-use quality with multi-trait genomic predictions incorporating near infrared and nuclear magnetic resonance-derived phenotypes. Theor. Appl. Genet. 130, 2505–2519. 10.1007/s00122-017-2972-7 28840266

[B27] Heffner E. L. Jannink J.-L. Iwata H. Souza E. Sorrells M. E. (2011). Genomic selection accuracy for grain quality traits in biparental wheat populations. Crop Sci. 51, 2597–2606. 10.2135/cropsci2011.05.0253

[B28] Hellin J. Shiferaw B. Cairns J. E. Reynolds M. Ortiz-Monasterio I. Banziger M.. (2012). Climate change and food security in the developing world: Potential of maize and wheat research to expand options for adaptation and mitigation. J. Dev. Agric. Econ. 4, 311–321. 10.5897/JDAE11.112

[B29] Heslot N. Akdemir D. Sorrells M. E. Jannink J. L. (2013). Integrating environmental covariates and crop modeling into the genomic selection framework to predict genotype by environment interactions. Theor. Appl. Genet. 127, 463–480. 10.1007/s00122-013-2231-5 24264761

[B30] Heslot N. Yang H.-P. Sorrells M. E. Jannink J.-L. (2012). Genomic selection in plant breeding: a comparison of models. Crop Sci. 52, 146–160. 10.2135/cropsci2011.06.0297

[B31] Jannink J.-L. Lorenz A. J. Iwata H. (2010). Genomic selection in plant breeding: from theory to practice. Brief. Funct. Genomics. 9, 166–177. 10.1093/bfgp/elq001 20156985

[B32] Jarquin D. Howard R. Crossa J. Beyene Y. Gowda M. Martini J. W. R.. (2020). Genomic prediction enhanced sparse testing for multi-environment trials. G3 Genes| Genomes|Genet. 10, 2725–2739. 10.1534/g3.120.401349 32527748 PMC7407457

[B33] Jarquín D. Silva C. L. da Gaynor R. C. Poland J. Fritz A. Howard R.. (2017). Increasing genomic-enabled prediction accuracy by modeling genotype × environment interactions in kansas wheat. Plant Genome 10:0130. 10.3835/plantgenome2016.12.0130 28724062

[B34] Juliana P. Montesinos-López O. A. Crossa J. Mondal S. González Pérez L. Poland J.. (2019). Integrating genomic-enabled prediction and high-throughput phenotyping in breeding for climate-resilient bread wheat. Theor. Appl. Genet. 132, 177–194. 10.1007/s00122-018-3206-3 30341493 PMC6320358

[B35] Juliana P. Singh R. P. Singh P. K. Crossa J. Huerta-Espino J. Lan C.. (2017a). Genomic and pedigree-based prediction for leaf, stem, and stripe rust resistance in wheat. Theor. Appl. Genet. 130, 1415–1430. 10.1007/s00122-017-2897-1 28393303 PMC5487692

[B36] Juliana P. Singh R. P. Singh P. K. Crossa J. Rutkoski J. E. Poland J. A.. (2017b). Comparison of models and whole-genome profiling approaches for genomic-enabled prediction of *Septoria Tritici Blotch, Stagonospora Nodorum Blotch*, and Tan Spot resistance in wheat. Plant Genome 10:0082. 10.3835/plantgenome2016.08.0082 28724084

[B37] Kassambara A. Mundt F. (2020). Factoextra: Extract and Visualize the Results of Multivariate Data Analyses. Available online at: https://cran.r-project.org/packagefactoextra (accessed May 05, 2020).

[B38] Kohavi R. (1995). A study of cross-validation and bootstrap for accuracy estimation and model selection. Proceedings of IJCAI'95 2, 1137–1143.

[B39] Lado B. Barrios P. G. Quincke M. Silva P. Gutiérrez L. (2016). Modeling genotype × environment interaction for genomic selection with unbalanced data from a wheat breeding program. Crop Sci. 56, 2165–2179. 10.2135/cropsci2015.04.0207

[B40] Lado B. Vázquez D. Quincke M. Silva P. Aguilar I. Gutiérrez L. (2018). Resource allocation optimization with multi-trait genomic prediction for bread wheat (*Triticum aestivum* L.) baking quality. Theor. Appl. Genet. 131, 2719–2731. 10.1007/s,00122-018-3186-3 30232499 PMC6244535

[B41] Lê S. Josse J. Husson F. (2008). FactoMineR: an R package for multivariate analysis. J. Stat. Softw. 25, 1–18. 10.18637/jss.v025.i01

[B42] Lillemo M. Ginkel M. van, Trethowan R. M. Hernandez E. Crossa J. (2005). Differential adaptation of CIMMYT bread wheat to global high temperature environments. Crop Sci. 45, 2443–2453. 10.2135/cropsci2004.0663

[B43] Lozada D. N. Mason R. E. Sarinelli J. M. Brown-Guedira G. (2019). Accuracy of genomic selection for grain yield and agronomic traits in soft red winter wheat. BMC Genet. 20, 1–12. 10.1186/s12863-019-0785-1 31675927 PMC6823964

[B44] Malosetti M. Bustos-Korts D. Boer M. P. Eeuwijk F. A. van (2016). Predicting responses in multiple environments: issues in relation to genotype × environment interactions. Crop Sci. 56, 2210–2222. 10.2135/cropsci2015.05.0311

[B45] Maphosa L. Langridge P. Taylor H. Parent B. Emebiri L. C. Kuchel H.. (2014). Genetic control of grain yield and grain physical characteristics in a bread wheat population grown under a range of environmental conditions. Theor. Appl. Genet. 7 127, 1607–1624. 10.1007/s00122-014-2322-y 24865506

[B46] Meuwissen T. H. E. Hayes B. J. Goddard M. E. (2001). Prediction of total genetic value using genome-wide dense marker maps. Genetics. 157. 4 1819–1829. 10.1093/genetics/157.4.1819 11290733 PMC1461589

[B47] Michel S. Kummer C. Gallee M. Hellinger J. Ametz C. Akgöl B.. (2018). Improving the baking quality of bread wheat by genomic selection in early generations. Theor. Appl. Genet. 131, 477–493. 10.1007/s00122-017-2998-x 29063161 PMC5787228

[B48] Mondal S. Singh R. P. Mason E. R. Huerta-Espino J. Autrique E. Joshi A. K. (2016). Grain yield, adaptation and progress in breeding for early-maturing and heat-tolerant wheat lines in South Asia. F. Crop. Res. 192, 78–85. 10.1016/j.fcr.2016.04.017 27307654 PMC4892352

[B49] Montesinos-López A. Montesinos-López O. A. Gianola D. Crossa J. Hernández-Suárez C. M. (2018). Multi-environment genomic prediction of plant traits using deep learners with dense architecture. G3 Genes|Genomes|Genet. 8, 3813–3828. 10.1534/g3.118.200740 30291107 PMC6288841

[B50] Norman A. Taylor J. Edwards J. Kuchel H. (2018). Optimising genomic selection in wheat: effect of marker density, population size and population structure on prediction accuracy. G3 Genes|Genomes|Genetics 8, 2889–2899. 10.1534/g3.118.200311 29970398 PMC6118301

[B51] Norman A. Taylor J. Tanaka E. Telfer P. Edwards J. Martinant J.-P.. (2017). Increased genomic prediction accuracy in wheat breeding using a large Australian panel. Theor. Appl. Genet. 130, 2543–2555. 10.1007/s00122-017-2975-4 28887586 PMC5668360

[B52] Oakey H. Cullis B. Thompson R. Comadran J. Halpin C. Waugh R. (2016). Genomic selection in multi-environment crop trials. G3 Genes|Genomes|Genet. 6, 1313–1326. 10.1534/g3.116.027524 26976443 PMC4856083

[B53] Olatoye M. O. Clark L. V. Labonte N. R. Dong H. Dwiyanti M. S. Anzoua K. G.. (2020). Training population optimization for genomic selection in miscanthus. G3 Genes|Genomes|Genet. 10, 2465–2476. 10.1534/g3.120.401402 32457095 PMC7341128

[B54] Pandey M. K. Chaudhari S. Jarquin D. Janila P. Crossa J. Patil S. C.. (2020). Genome-based trait prediction in multi- environment breeding trials in groundnut. Theor. Appl. Genet. 133, 3101–3117. 10.1007/s00122-020-03658-1 32809035 PMC7547976

[B55] Pérez-Rodríguez P. Gianola D. González-Camacho J. M. Crossa J. Manès Y. Dreisigacker S. (2012). Comparison between linear and non-parametric regression models for genome-enabled prediction in wheat. G3 Genes|Genomes|Genetics 2, 1595–1605. 10.1534/g3.112.003665 23275882 PMC3516481

[B56] Poland J. A. Brown P. J. Sorrells M. E. Jannink J.-L. (2012). Development of high-density genetic maps for barley and wheat using a novel two-enzyme Genotyping-by-Sequencing approach. PLoS ONE 7:e32253. 10.1371/journal.pone.0032253 22389690 PMC3289635

[B57] Poland J. A. Rife T. W. (2012). Genotyping-by-Sequencing for plant breeding and genetics. Plant Genome 5:005. 10.3835/plantgenome,2012.05.0005

[B58] R Core Team (2019). R: A Language and Environment for Statistical Computing. R Found. Stat. Comput. Avaialble online at: https://www.R-project.org/

[B59] Rincent R. Kuhn E. Monod H. Oury F.-X. Rousset M. Allard V.. (2017). Optimization of multi-environment trials for genomic selection based on crop models. Theor. Appl. Genet. 130, 1735–1752. 10.1007/s00122-017-2922-4 28540573 PMC5511605

[B60] Rode J. Ahlemeyer J. Friedt W. Ordon F. (2011). Identification of marker-trait associations in the German winter barley breeding gene pool (*Hordeum vulgare* L.). Mol. Breed. 30, 831–843. 10.1007/s11032-011-9667-6

[B61] Roorkiwal M. Jarquin D. Singh M. K. Gaur P. M. Bharadwaj C. Rathore A.. (2018). Genomic-enabled prediction models using multi-environment trials to estimate the effect of genotype × environment interaction on prediction accuracy in chickpea. Sci. Rep. 8, 1–11. 10.1038/s41598-018-30027-2 30076340 PMC6076323

[B62] Rutkoski J. Singh R. P. Huerta-Espino J. Bhavani S. Poland J. Jannink J. L.. (2015). Genetic gain from phenotypic and genomic selection for quantitative resistance to stem rust of wheat. Plant Genome 8:74. 10.3835/plantgenome2014.10.0074 33228306

[B63] Sallam A. H. Endelman J. B. Jannink J.-L. Smith K. P. (2015). Assessing genomic selection prediction accuracy in a dynamic barley breeding population. Plant Genome 8:20. 10.3835/plantgenome2014.05.0020 33228279

[B64] Sehgal D. Autrique E. Singh R. Ellis M. Singh S. Dreisigacker S. (2017). Identification of genomic regions for grain yield and yield stability and their epistatic interactions. Sci. Rep. 7, 1–12. 10.1038/srep41578 28145508 PMC5286416

[B65] Sharma R. C. Smith E. L. (1986). Selection for high and low harvest index in three winter wheat populations1. Crop Sci. 26, 1147–1150. 10.2135/cropsci1986.0011183X002600060013x

[B66] Thorwarth P. Ahlemeyer J. Bochard A.-M. Krumnacker K. Blümel H. Laubach E.. (2017). Genomic prediction ability for yield-related traits in German winter barley elite material. Theor. Appl. Genet. 130, 1669–1683. 10.1007/s00122-017-2917-1 28534096

[B67] Tolhurst D. J. Mathews K. L. Smith A. B. Cullis B. R. (2019). Genomic selection in multi-environment plant breeding trials using a factor analytic linear mixed model. J. Anim. Breed. Genet. 136, 279–300. 10.1111/jbg.12404 31247682

[B68] Tomar V. Dhillon G. S. Singh D. Singh R. P. Poland J. Chaudhary A. A.. (2021). Evaluations of genomic prediction and identification of new loci for resistance to stripe rust disease in wheat (Triticum aestivum L.) Front. Genet. 12:710485 (in press). 10.3389/fgene.2021.710485 34650592 PMC8505882

[B69] Velu G. Crossa J. Singh R. P. Hao Y. Dreisigacker S. Perez-Rodriguez P.. (2016). Genomic prediction for grain zinc and iron concentrations in spring wheat. Theor. Appl. Genet. 129, 1595–1605. 10.1007/s00122-016-2726-y 27170319

[B70] Wang X. Li L. Yang Z. Zheng X. Yu S. Xu C.. (2016). Predicting rice hybrid performance using univariate and multivariate GBLUP models based on North Carolina mating design II. Hered 118, 302–310. 10.1038/hdy.2016.87 27649618 PMC5315526

[B71] Würschum T. Leiser W. L. Langer S. M. Tucker M. R. Longin C. F. H. (2018). Phenotypic and genetic analysis of spike and kernel characteristics in wheat reveals long-term genetic trends of grain yield components. Theor. Appl. Genet. 131, 2071–2084. 10.1007/s00122-018-3133-3 29959471

[B72] Zhang J. Song Q. Cregan P. B. Jiang G.-L. (2015). Genome-wide association study, genomic prediction and marker-assisted selection for seed weight in soybean (Glycine max). Theor. Appl. Genet. 129, 117–130. 10.1007/s00122-015-2614-x 26518570 PMC4703630

