# Supplementary figures and images for: Increased Predictive Accuracy of Multi-Environment Genomic Prediction Model for Yield and Related Traits in Spring Wheat (Triticum aestivum L.)
Source: Front Plant Sci. 2021 Oct 8;12:720123. doi: 10.3389/fpls.2021.720123 (PMC8531512; doi:10.3389/fpls.2021.720123)

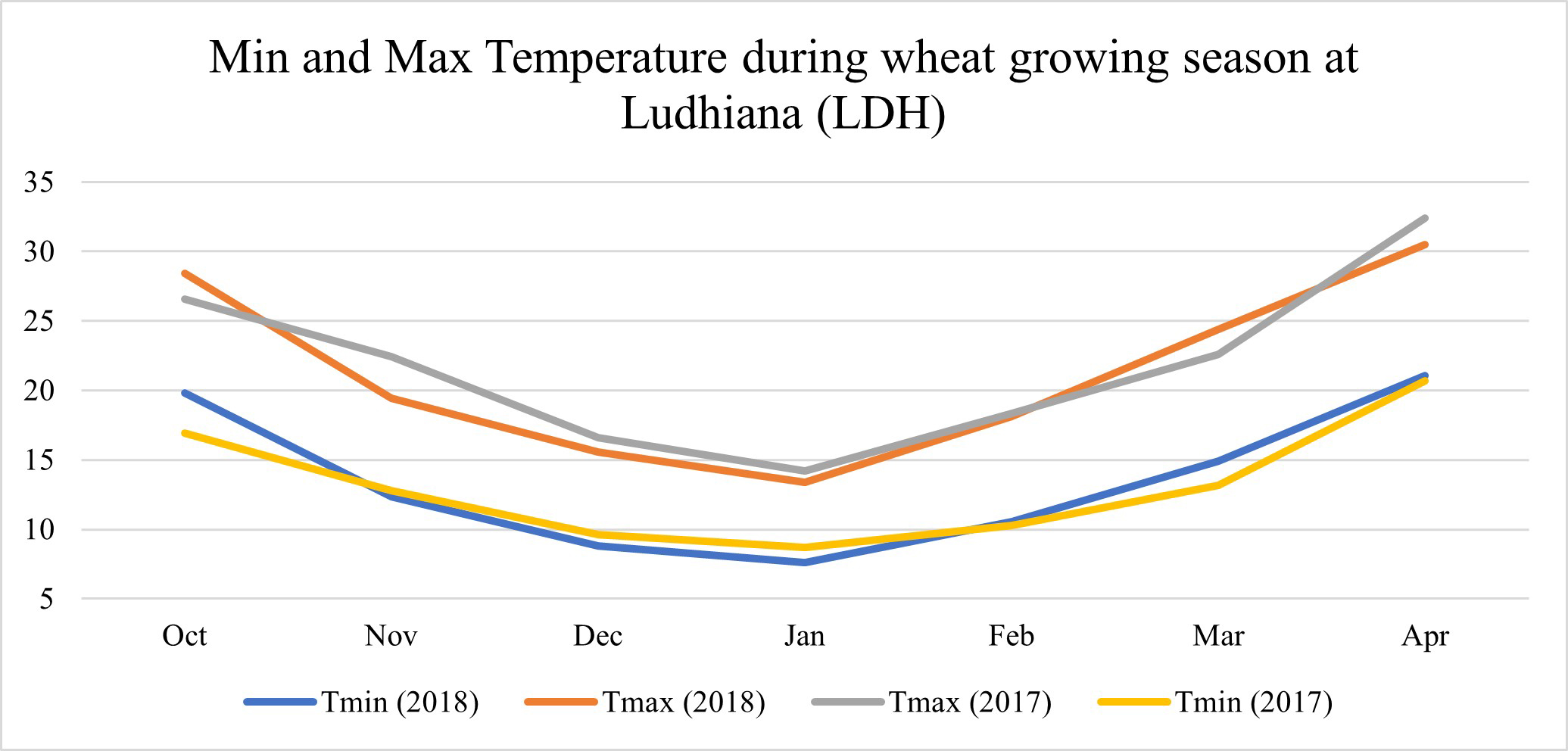

Supplement: Supplementary Figure 1 — Weather information of LDH17 and LDH18. [file Image_1.JPEG]

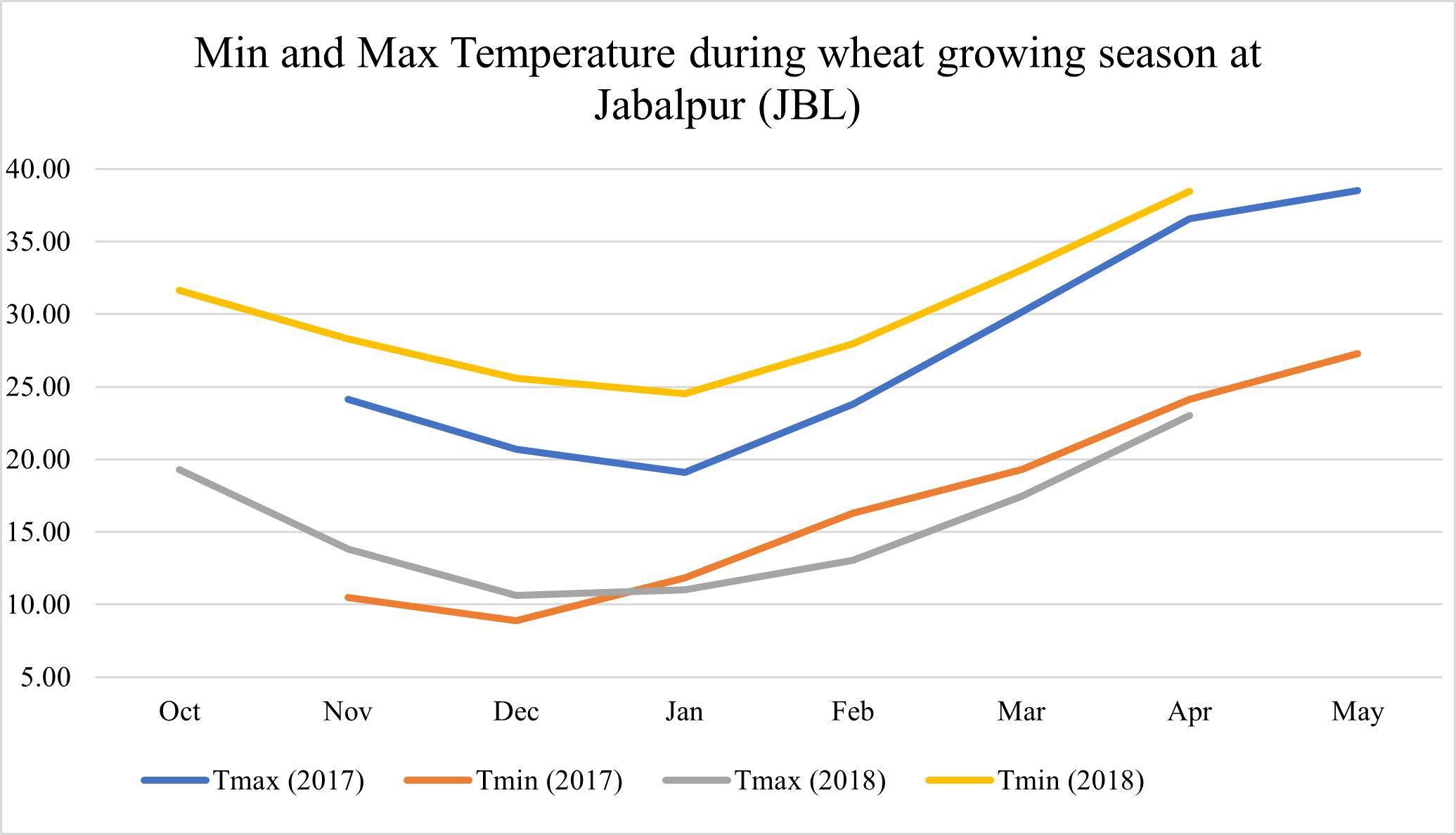

Supplement: Supplementary Figure 2 — Weather information of JBL17 and JBL18. [file Image_2.JPEG]
