# Supplementary material for: Increased Predictive Accuracy of Multi-Environment Genomic Prediction Model for Yield and Related Traits in Spring Wheat (Triticum aestivum L.)
Source: Front Plant Sci. 2021 Oct 8;12:720123. doi: 10.3389/fpls.2021.720123 (PMC8531512; doi:10.3389/fpls.2021.720123)
Supplement: Supplementary Table 2 — List of traits that were evaluated during this study in the field trials. [file Table_2.DOCX]

**Table S2**: List of traits that were evaluated during this study in the field trials.

| **Trait** | **Abbreviation** | **Unit of measurement** | **Description** |
| --- | --- | --- | --- |
| Days to Maturity | DAYSMT | days | Maturity time starts at the hard dough stage (ds87) often called physiological maturity |
| Heading Date | DTHD | days | Heading time extends from the time of emergence of the tip of the spike from the flag leaf sheath to when the spike has completely emerged but has not yet started to flower |
| Grain Yield | GRYLD | tons/hectare (t/ha) | Weight of grains that were harvested |
| Thousand Grain Weight | TGW | grams (g) | Grain weight expressed as the weight of thousand grains |
